# PPFIBP1 induces glioma cell migration and invasion through FAK/Src/JNK signaling pathway

**DOI:** 10.1038/s41419-021-04107-7

**Published:** 2021-09-03

**Authors:** Caihua Dong, Xinying Li, Jiao Yang, Detian Yuan, Yuanshuai Zhou, Yina Zhang, Guohua Shi, Ruobing Zhang, Jianping Liu, Peng Fu, Minxuan Sun

**Affiliations:** 1 School of Biomedical Engineering (Suzhou), Division of Life Sciences and Sciences and Medicine, University of Science and Technology of China, Hefei, 230026 China; 2 Jiangsu Key Laboratory of Medical Optics, Suzhou Institute of Biomedical Engineering and Technology, Chinese Academy of Sciences, Suzhou, 215163 China; 3 Department of Biochemistry and Molecular Biology, School of Basic Medical Sciences, Cheeloo College of Medicine, Shandong University, Jinan, 250012 China; 4 Neurological Department, Helios-Amper Clinic Dachau, Dachau, Germany; 5 Integrated Cardio Metabolic Centre, Karolinska Institute, Huddinge, Sweden; 6 Department of Neurosurgery, Union Hospital, Tongji Medical College, Huazhong University of Science and Technology, Wuhan, 430022 China

**Keywords:** Oncogenes, Molecular neuroscience

## Abstract

Glioblastoma multiforme (GBM) is the most aggressive brain tumor, with a 5-year survival ratio <5%. Invasive growth is a major determinant of the poor prognosis in GBM. In this study, we demonstrate that high expression of PPFIA binding protein 1 (PPFIBP1) correlates with remarkable invasion and poor prognosis of GBM patients. Using scratch and transwell assay, we find that the invasion and migration of GBM cells are promoted by overexpression of PPFIBP1, while inhibited by knockdown of PPFIBP1. Then, we illustrate that overexpression of PPFIBP1 facilitates glioma cell infiltration and reduces survival in xenograft models. Next, RNA-Seq and GO enrichment analysis reveal that PPFIBP1 regulates differentially expressed gene clusters involved in the Wnt and adhesion-related signaling pathways. Furthermore, we demonstrate that PPFIBP1 activates focal adhesion kinase (FAK), Src, c-Jun N-terminal kinase (JNK), and c-Jun, thereby enhancing Matrix metalloproteinase (MMP)-2 expression probably through interacting with SRCIN1 (p140Cap). Finally, inhibition of phosphorylation of Src and FAK significantly reversed the augmentation of invasion and migration caused by PPFIBP1 overexpression in GBM cells. In conclusion, these findings uncover a novel mechanism of glioma invasion and identify PPFIBP1 as a potential therapeutic target of glioma.

## Introduction

Gliomas are the most common aggressive primary brain tumors, of which glioblastoma multiforme (GBM) is the most malignant form (WHO grade IV) in human. GBM accounts for 50% of all primary brain gliomas [1]. Despite combination treatment of maximal safe surgical resection, radiation, and chemotherapy, the median survival of GBM patients is <15 months [2, 3]. Diffuse invasion is the major obstacle to cure GBM [4, 5], as invasive GBM cells are more resistant to current chemo- and radiation-therapies, and thus prone to escape from complete surgical resection [6]. Therefore, understanding the molecular mechanism of GBM invasion will be crucial to identify potential therapeutic targets.

The past decade has witnessed essential progress in understanding of glioma cell invasion. Aggressive glioma cells vigorously migrate through the tortuous extracellular spaces of the brain, resulting in the formation of distant satellite tumors [5]. In general, cell migration involves four distinct steps: (1) leading edge protrusion, (2) turnover of focal adhesions, (3) generation of tractional forces, and (4) tail retraction and detachment [7–9]. Previous studies have revealed that Src and FAK promoted focal adhesion turnover during transformation and migration, which is indispensable for cell migration on extracellular matrix [10–12].

The LAR protein-tyrosine phosphatase-interacting protein (liprin) family plays key roles in lymphatic vessel integrity, active zone formation, and pre- and post-synaptic development [13, 14]. The mammalian liprin family mainly consists of four liprin-α (liprin-α1 to -α4) proteins and two liprin-β (-β1 and -β2) proteins. Liprin-α and liprin-β form heterodimers and act as scaffolds together [15]. Previous studies revealed different roles of liprins in cancer progress, especially in cell motility and migration [16].

Liprin-β1, also named PPFIBP1, is one of the ubiquitously expressed liprins. Evidence suggests that PPFIBP1 promotes cell motility and migration in breast cancer and melanoma [16, 17]. PPFIBP1 has strong association with Kank1 and Kank2 proteins, which are involved in suppression of cellular proliferation and regulation of cell migration in melanoma [17]. PPFIBP1 interacts with metastasis-associated protein S100A4, which induces invasiveness of primary tumors and promotes metastasis in melanoma [18]. Recent study also demonstrated an association between elevated PPFIBP1 expression and tumor malignancy in liver cancer [19]. However, its potential role in GBM and the mechanism by which it promotes cell migration and invasion remains incompletely understood.

In this study, we illustrate that the expression of PPFIBP1 positively correlates with tumor invasion and the prognosis of glioma patients. Our data suggest that elevation of PPFIBP1 expression in glioma leads to increased integrin α3, integrin α4, and integrin β8 and MMP-2 expression, along with enhanced cell migration and invasion via activation of FAK/Src/JNK signaling pathway, probably through interacting with SRCIN1.

## Materials and methods

### Reagents and antibodies

DMSO, Tween-20, Anti-FLAG M2 Magnetic Beads, and puromycin were purchased from Sigma-Aldrich (Shanghai, China). Dasatinib and PF562711 were obtained from Selleck Chemicals (Shanghai, China) and dissolved in DMSO. Fetal bovine serum (FBS), penicillin, and streptomycin were purchased from Gibco (South America). DMEM and MEM medium were purchased from Hyclone (Logan, Utah, USA). Nitrocellulose blotting membrane was obtained from GE Healthcare. Matrigel was provided by BD Biosciences (USA). Protein G agarose was purchased from Roche. 3×FLAG Peptide was purchased from APExBIO (USA). Silver staining kit was obtained from Beyotime Biotechnology (Shanghai, China). Antibodies used in this study are listed in Supplementary Table 1.

### Cell culture, lentivirus production, and transduction

U87 MG and U251 MG were obtained from the cell bank of type culture collection of Chinese Academy of Sciences and verified by short tandem repeat assays for their identifications. Monthly mycoplasma tests were performed by PCR. Cells were thawed from the original stocks and cultured at 37 °C under 5% CO_2_ for <3 weeks until further use. U251 MG cells and U87 MG cells were cultivated in DMEM medium and in MEM medium, respectively, supplemented with 1% Penicillin-Streptomycin and 10% FBS.

Short hairpin interfering RNA sequences (Supplementary Table 2) were cloned into pPLK-GFP-Puro, while cDNA of PPFIBP1 was cloned into pLVX-GFP-Puro lentiviral vector. Lentiviral plasmids, envelope plasmid (PSPAX2), and gag-pol plasmid (PMD2G) were transfected together into HEK293T cells with FuGENE HD transfection reagent (Promega, USA) to produce viruses. 48 and 72 h after transfection, culture media was collected and filtered with 0.45-mm filter.

Briefly, U87 MG and U251 MG cells were seeded into 6-well plates (1 × 10^5^ cells per well). After 24 h culture, cells were incubated with viral supernatants for 24 h in the presence of 8 μg/ml polybrene (Hanbio Biotechnology Co. Ltd, China). Two days after infection, transduced cells were selected with 2 μg/ml puromycin (Beyotime Biotechnology, China) for 3 days.

### Matrigel transwell assay

The invasion assay was performed according to the manufacturer (Costar, Cambridge, USA). We used modified Boyden chambers with 8-μm pore filter inserts for 24-well plate (Costar). The filters were pre-coated with 100 μl ice-cold 10% Matrigel in cold DMEM. Then, 1 × 10^5^ cells in 200 μl serum-free medium were added to the upper chamber and 600 μl complete medium was placed in the lower chamber. Cells were incubated at 37 °C under 5% CO_2_ for 20 h and then fixed with 4% paraformaldehyde for 10 min. Cells remained on the upper surface were removed with cotton swabs. Cells that had migrated through the filter were stained with 0.5% crystal violet solution for 10 min. Images of the stained cells were captured using a Nikon compound microscope. Four representative fields per membrane were imaged. The cell number of each field were counted. Experiments were independently repeated three times.

### Wound-healing assay

Cells were seeded in 6-well plate (4 × 10^5^ cells per well) and incubated for 12 h to achieve 90–100% confluence. Then, cells were cultured in DMEM or MEM medium supplemented with 1% FBS for 12 h. Wounds were gently made by scratching with a 200 μl pipette tip. After washing with phosphate-buffered saline (PBS) two times, cells were further cultured in DMEM or MEM medium supplemented with 1% FBS at 37 °C. The wounds were imaged every 10 h. Assays were repeated three times for each cell line. The cell migration activity was quantified by width of wound area at different time points relative to the original width of wound.

### Human glioma samples

Clinical glioma specimens from 47 patients were collected in Wuhan Union Hospital and processed to the research laboratories after de-identification of the samples, as described previously [20]. About 51% (24/47) of the surgery cases were low-grade glioma (LGG) and 49% (23/47) were GBM. Moreover, combining the histological and radiological characteristics (enhanced MRI images) [21, 22], the 23 LGG samples were further classified into noninvasive (*n* = 13) and invasive (*n* = 10) by two independent neuropathologists. All samples were collected with signed informed consent according to the internal review and ethics boards of Wuhan Union Hospital. The procedure was approved by the ethics boards of Wuhan Union Hospital.

### Cell line-derived xenograft (CDX) models

Male BALB/c nude mice of 8- to 10-week-old age were purchased from SiPeiFu Biotechnology Co., Ltd. (Beijing), housed under standard conditions at the animal care facility at Center of Experimental Animal of Suzhou Institute of Biomedical Engineering and Technology, Chinese Academy of Sciences. Briefly, using a stereotactic frame, 1 × 10^5^ GFP expressing glioma cells were planted into the striatum area (−2.0; 1.0; −3.0). Eight mice were used for each group. When related neuropathological symptoms developed, mice were sacrificed and perfused with PBS and 4% paraformaldehyde (PFA). Mice brains were dissected, fixed in 4% PFA for 24 h. The procedure has been approved by Animal Care and Use Committee, Suzhou Institute of Biomedical Engineering and Technology, Chinese Academy of Sciences.

### Quantification of tumor protrusions of intracranial tumor

Cryosections (40 μm) were performed with H&E staining, and then captured using a Nikon compound microscope. Regions of interest were selected at the interface of tumor center and infiltrative edge. After staining with DAPI, cryosections with GFP expressing glioma cells were imaged with Olympus VS120. Invading cells of the jagged tumor periphery form micro tumor protrusions [23]. The invasion of tumor cell was measured by quantifying the number of micro tumor protrusions of infiltrating edge. At least three distinct tumor sections located in different regions of the tumor were analyzed for each group.

### Immunohistochemical (IHC) staining

For paraffin slides from tissue of patients, after deparaffinization and hydration, slides were boiled in Tris-EDTA buffer (PH = 8.0) for 10 min for antigen retrieval, then washed with PBS; while frozen tissue slides of xenograft were washed with PBS for three times. Next, sections were treated with 3% H_2_O_2_ for 20 min to bleach endogenous peroxidase. After blocking with donkey serum in PBS for 30 min, slides were incubated at 4 °C overnight with primary antibodies (Supplementary Table 1). After wash with PBS, slides were incubated with HRP-conjugated donkey anti-rabbit or mouse secondary antibody (DAKO) for 45 min at room temperature, followed by staining by DAB with Gill hematoxylin counterstaining. Samples incubated without primary antibodies were used as negative controls. The expression of PPFIBP1 in human glioma specimens was scored as 0 (absent), 1 (weak), 2 (moderate), and 3 (strong) in a double-blinded manner. Patients were accordingly stratified into PPFIBP1^Low^ (score of 0–1) versus PPFIBP1^High^ (score of 2–3) groups.

### Immunoprecipitation and western blot

For co-immunoprecipitation (Co-IP), cells were lysed using Co-IP lysis buffer (20 mmol/L Tris (pH 7.5), 150 mmol/L NaCl, and 1% Triton X-100) supplemented with a protease and phosphatase inhibitor cocktail. The supernatants of the lysates were collected after centrifugation and then incubated with the indicated antibodies overnight at 4 °C with constant rotation. Then, the antibodies in the lysates were precipitated with protein A/G magnetic beads (Millipore) and washed with PBS. And for western blot analysis, cell lysates were prepared in RIPA lysis buffer including protease inhibitors (CoWin Biosciences, China) and phosphatase inhibitors (CoWin Biosciences, China). Equal amounts of proteins were subjected to SDS-polyacrylamide gels and transfer to nitrocellulose membranes. Subsequently, the membrane was blocked with 5% non-fat dried milk for 1 h at room temperature and then incubated with the primary antibodies overnight at 4 °C. After incubation with IRDye 800cw or 680cw conjugated antibodies (1:10,000 dilution) for 1 h, the membranes were imaged with Odyssey® CLx Infrared Imaging System.

### Real-time quantitative PCR

Total RNA was isolated using RNAiso Plus (Takara, China) and reverse transcribed with Rever Tra Ace qPCR RT Master Mix (EZBioscience, China). The cDNAs were then used for real-time PCR (RT-qPCR) on a 7500 Fast Real-Time PCR System (Applied Biosystems) using SYBR Green Realtime PCR Master (Toyobo, China). GAPDH served as an internal control. The relative quantity of gene expression was analyzed by the 2^−ΔΔCt^ method. The primers used for qPCR analyses are listed in Supplementary Table 2.

### RNA-sequencing analysis

The total RNAs from PPFIBP1 overexpressed or silenced U87 MG cells and corresponding NC cells were isolated and underwent quality control. The preparation of whole transcriptome libraries and deep sequencing were performed by Novogene Bioinformatics Technology Cooperation (Beijing, China). Raw reads were mapped to the homo sapiens reference genomics (hg19) with HISAT2 software [24]. FeactureCounts was employed for quantification of mapped reads into genomic features [25]. The differential expression analysis was performed by limma package [26], the genes with fold change >2, ajusted *P* value < 0.05, were identified as differentially expressed genes (DEGs). To assess the functional features of differentially expressed genes, clusterProfiler and GSEABase were applied for functional annotation [27] and enrichment analysis [28], respectively. The functional terms with adjusted *P* value < 0.05 were identified as correlated terms.

### LC–MS/MS analysis

Co-IP was performed using FLAG M2 beads as described above. Proteins were eluted with 200 μg/ml 3×FLAG-peptide in PBS for 30 min. Immunoprecipitation samples were separated by sodium dodecyl sulfate–polyacrylamide gel electrophoresis, and visualized with silver staining. As previously described [29, 30], LC–MS/MS analysis was performed by Guangzhou Fitgene Biotechnology Co. Briefly, the gel was cut into slices, proteins were digested in gel with trypsin, and the residing peptides were extracted and lyophilized for further analysis. Peptides were suspended in 2% acetonitrile and 0.1% formic acid. For the LC run, samples were loaded onto a 75 μm i.d. × 150 mm reverse-phase column, packed with Acclaim PepMap RSLC C18. Separated peptides were directly analyzed with the mass spectrometer (Thermo Scientific Q Exactive) for online detection. The resulting spectra were recorded for each run. MS data were searched on Sorcerer2-SEQUEST using the reviewed Swiss-Prot database.

### Statistical analysis

All data are presented as means ± SEM. Statistical differences between two groups were evaluated using a two-tailed *t*-test. Overall survival (OS) was plotted by the Kaplan–Meier method and compared by the log-rank test. Statistical analysis was performed using GraphPad Prism 7.0. Statistical significance was defined as: *P < 0.05, **P < 0.01, ***P < 0.001.

## Results

### High expression of PPFIBP1 in glioma correlates with increased invasion and poor prognosis of patients

**Fig. 1:**
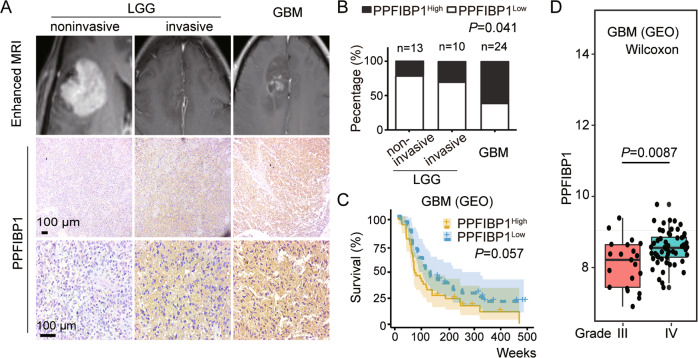
PPFIBP1 expression and its correlation with invasion and survival in glioma patients. **A** Top: representative MRI images of patients with LGG and GBM. Middle and bottom: low and high magnification of glioma tumor sections of patient stained with a PPFIBP1 antibody. **B** PPFIBP1 expression levels in glioma specimens from 47 glioma patients. Statistical difference between LGG noninvasive, LGG invasive, and GBM was evaluated with Fisher’s exact test. **C** Kaplan–Meier curves show the overall survival of 77 GBM patients, of which 38 are with high level of PPFIBP1 and 39 are with low level of PPFIBP1. Data were obtained from GEO database (GSE4271). **D** The boxplot shows the expression levels of PPFIBP1 in patients with different grades of the same data set from GEO database (GSE4271), *P* value is determined by Wilcoxon signed-rank test. The figures are representative data from at least three independent experiments.

Immunohistochemical (IHC) staining on tumor tissues from patients were performed to investigate the relationship between PPFIBP1 protein levels and the aggressiveness of glioma. Patients were accordingly stratified into PPFIBP1^Low^ (score of 0–1) versus PPFIBP1^High^ (score of 2–3) groups. The results indicated that PPFIBP1 protein levels in GBM tissues (14/23, 61%) were significantly higher than that in LGG (6/24, 25%) (*P* = 0.041, Fig. 1A), and higher in invasive LGG tissues (3/10, 30%) than in noninvasive LGG tissues (3/14, 21%), although not statistically significant (Fig. 1B). The Cancer Genome Atlas (TCGA) and Gene Expression Omnibus (GEO, GSE4271) database analysis demonstrated that glioma group with high PPFIBP1 expression displayed shorter survival than that with low PPFIBP1 expression, although not statistically significant (*P* = 0.057, Fig. 1C and Figure S1A). Furthermore, the GEO (GSE4271) database analysis also indicated that transcriptional level of PPFIBP1 in tissues from patients with grade IV was higher than that in grade III (*P* = 0.0087) (Fig. 1D). Similarly, the TCGA database analysis showed that the transcriptional levels of PPFIBP1 in mesenchymal and proneural subtype were significantly higher than in classical subtype (*P* = 0.0078; *P* = 0.003) (Figure S1B). These findings indicated that PPFIBP1 was highly positively correlated with glioma invasion and disease progression of glioma patients.

### PPFIBP1 overexpression promotes glioma cell migration and invasion

**Fig. 2:**
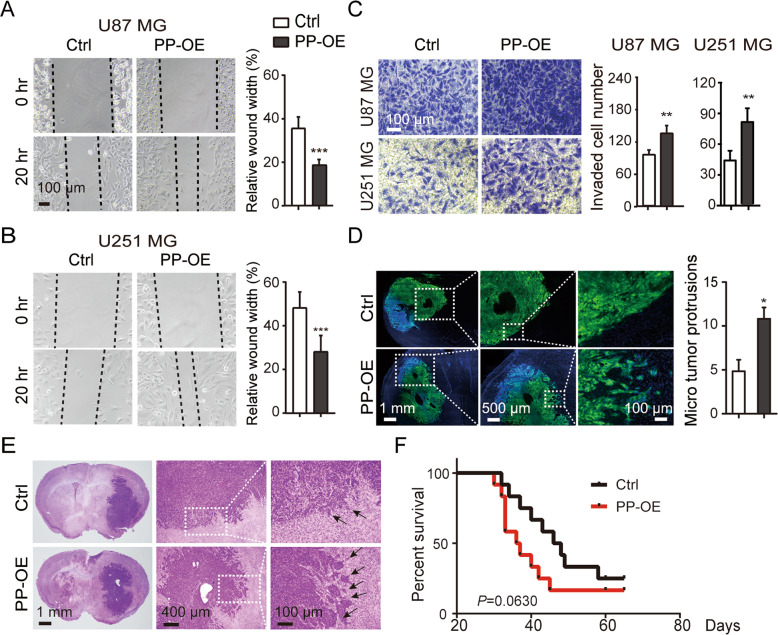
PPFIBP1 overexpression promotes glioma cell migration and invasion in vitro and in vivo. **A**, **B** Wound-healing assays of U87 MG and U251 MG derived cells with PPFIBP1 overexpression (PP-OE) and vector control (Ctrl) at indicated time points. Left: representative bright-field pictures of cells at 0 or 20 h. Dashed line represents the wound edge. Right: relative quantification of the (20 h/0 h) wound width. **C** Transwell invasion assay of U87 MG and U251 MG derived cells with PPFIBP1 overexpression (PP-OE) and vector control (Ctrl). Left: Crystal violet-stained migrated cells. Right: Number of invaded cells through the membrane per field, the black and white columns represent the PP-OE and the Ctrl group. **D** Representative images of xenografts with GFP expression glioma cells and quantification of micro tumor protrusions per field. The black and white columns represent the PP-OE and the Ctrl group. **E** Representative H&E stained images of mouse brain sections. The black arrow indicates the micro tumor protrusions. **F** Kaplan–Meier survival curves show the survival differences of tumor-bearing mice. The figures are representative data from at least three independent experiments. The data represent the mean ± SEM (*n* ≥ 3). Statistical analysis was performed using Student’s *t*-test. *P < 0.05, **P < 0.01, and ***P < 0.001.

To examine the role of PPFIBP1 in GBM cells, human PPFIBP1 was stably overexpressed in the U87 MG and U251 MG cell lines by lentiviral vector. The overexpression efficiency was verified by both RT-qPCR and western blotting (Figure S2E). The CellTiter Blue Assay revealed that PPFIBP1 overexpression has no significant effect on cell proliferation (Figure S2A–D). Wound-healing assay and transwell assay were performed to assess the role of PPFIBP1 on the migration and invasion of glioma cells. We found that PPFIBP1-overexpressing (PP-OE) glioma cells migrated faster than the vector control (Ctrl) cells (Fig. 2A, B), with increased invasion activity (Fig. 2C).

Next, the role of PPFIBP1 in glioma cells migration was confirmed using a mice intracranial glioma model. PP-OE U251 MG cells and vector control cells was inoculated into the striatum area of nude mice. Mouse brains were harvested for further analysis when neuropathological symptoms (hunched back and be lethargic) developed. Brains of PP-OE group developed more micro tumor protrusions compared with those of the Ctrl group (Fig. 2D, E). In addition, a shorter survival time was observed in the PP-OE group compared with that in the Ctrl group, although not statistically significant (Fig. 2F). Together, these findings demonstrated that overexpression of PPFIBP1 promotes glioma cell migration and invasion in vitro and in vivo.

### Knockdown of PPFIBP1 inhibits glioma cell migration and invasion in vitro and in vivo

**Fig. 3:**
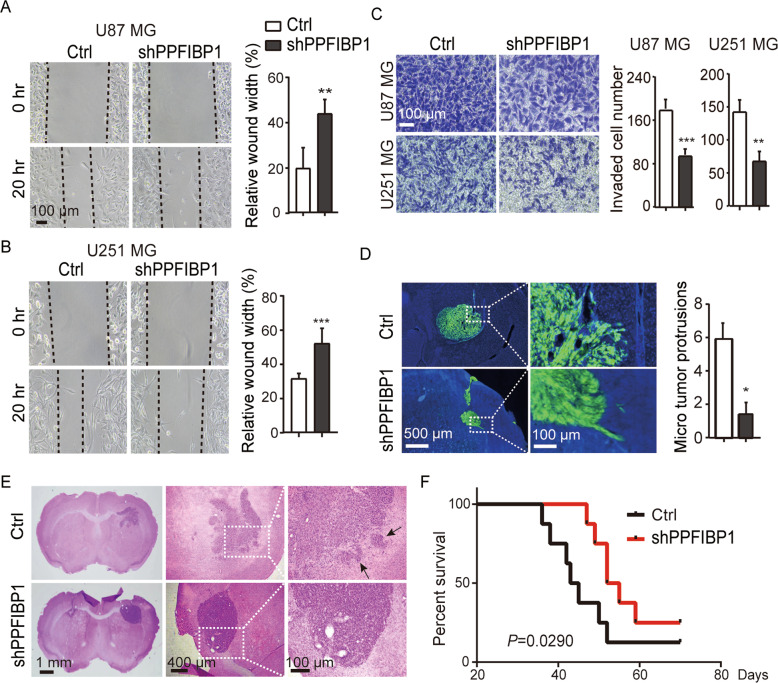
Knockdown of PPFIBP1 inhibits glioma cell migration and invasion in vitro and in vivo. **A**, **B** Wound-healing assays of U87 MG and U251 MG derived cells with PPFIBP1 knockdown (shPPFIBP1) and vector control (Ctrl) at indicated time points. Left: representative bright-field pictures of cells at 0 or 20 h. Right: relative quantification of the (20 h/0 h) wound width. Dashed line represents the wound edge. **C** Transwell invasion assay of U87 MG and U251 MG derived cells with PPFIBP1 knockdown (shPPFIBP1) and vector control (Ctrl). Left: Crystal violet-stained migrated cells. Right: Number of invaded cells through the membrane per field, the black and white columns represent the shPPFIBP1 and the Ctrl group. **D** Representative images of xenografts with GFP expressing glioma cells and quantification of micro tumor protrusions per field. The black and white columns represent the shPPFIBP1 and the Ctrl group. **E** Representative H&E stained images of mouse brain sections. The black arrow indicates the micro tumor protrusions. **F** Kaplan–Meier survival curves show the survival differences of tumor-bearing mice. The figures are representative data from at least three independent experiments. The data represent the mean ± SEM (*n* ≥ 3). Statistical analysis was performed using Student’s *t*-test. *P < 0.05, **P < 0.01, and ***P < 0.001.

To confirm the effect of PPFIBP1 overexpression on GBM cell migration and invasion, we also generated two small hairpin RNAs targeting PPFIBP1 in glioma cells. Knockdown (KD) efficiency (up to 75%) was verified by RT-qPCR and western blotting (Figure S1E), and subsequently we chose one (shPPFIBP1) in the following experiments. As expected, the PPFIBP1 KD glioma cells migrated slower than the Ctrl cells and showed decreased invasive capacity (Fig. 3A–C). Similarly, orthotropic glioma mouse model was also developed with PPFIBP1 KD and the Ctrl U251 MG cells. Brains of shPPFIBP1 group developed less micro tumor protrusions compared with the Ctrl group (Fig. 3D, E). Moreover, a prolonged survival time was observed in the PPFIBP1 KD group (Fig. 3F). Altogether, these results suggest that silencing of PPFIBP1 diminishes glioma cell migration and invasion in vitro and in vivo.

### PPFIBP1 regulates focal adhesion pathway of glioma cells

**Fig. 4:**
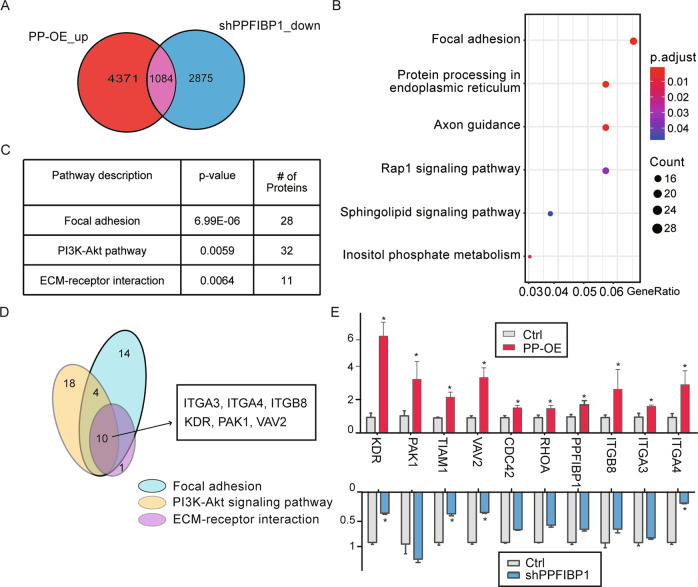
PPFIBP1 regulates focal adhesion pathway. **A** Venn diagram represents genes upregulated in PPFIBP1-OE cells and genes downregulated in PPFIBP1 knockdown cells. **B** KEGG pathway analysis of DEGs regulated by PPFIBP1. **C** Gene ontology enrichment analysis of DEGs regulated by PPFIBP1. **D** Venn diagram represents DEGs of the top three pathways. **E** Relative expression of the overlapped DEGs in (**D**) were analyzed by RT-qPCR. The data represents the mean ± SEM (*n* ≥ 3). Statistical analysis was performed using Student’s *t*-test. *P < 0.05.

We next explored the underlying mechanisms by comparing the transcriptomics of PP-OE, PPFIBP1 KD, and the Ctrl cells through RNA sequencing (RNA-seq) (Fig. 4A). There was more than 25% overlap between upregulated genes in PP-OE cells and downregulated genes in PPFIBP1 silenced cells. Gene ontology enrichment and KEGG pathway analyses of the differentially expressed genes (DEGs) revealed that these genes were clustered into the Wnt and focal adhesion-related signaling pathways (Fig. 4B, C). These results are in line with the analysis of clustered proteins with DAVID tool, suggesting that PPFIBP1 may be involved in cell adhesion [31]. We present here ten overlapped DEGs from the top three regulated signaling pathways (Fig. 4D). RT-qPCR of these DEGs confirmed that PPFIBP1 regulates multiple adhesion-related genes such as integrins (ITGA3, ITGA4, ITGB8), KDR, PAK1, and VAV2 (Fig. 4E). Taking together, these data demonstrated that PPFIBP1 might promotes glioma cells migration and invasion through regulating focal adhesion pathway.

### PPFIBP1 may enhance the phosphorylation of FAK and Src through interacting with SRCIN1

**Fig. 5:**
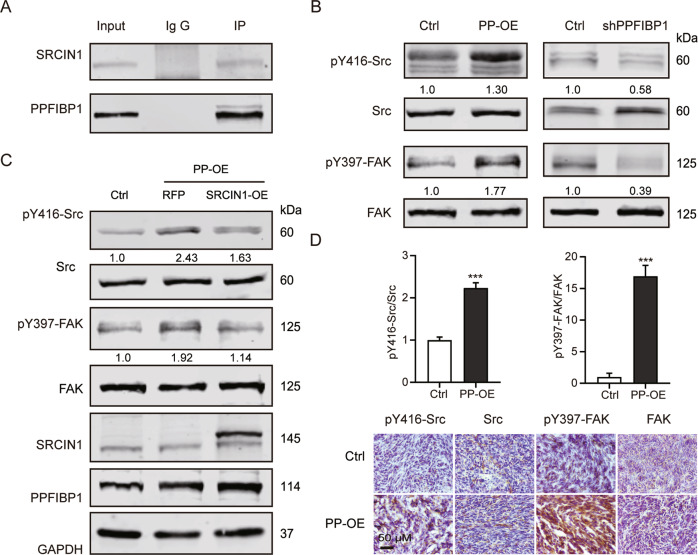
PPFIBP1 may enhance the phosphorylation of FAK and Src through interacting with SRCIN1. **A** Co-IP was performed, using an anti-PPFIBP1 antibody with non-specific IgG as negative control, followed by immunoblotting for indicated proteins. **B** Western blot showing the indicated proteins in PPFIBP1 overexpression and knockdown U251 MG cells. The numbers represent the expression of pY397 FAK, normalized to FAK, and the expression of pY416 Src, normalized to Src. The figures are representative data from at least three independent experiments. **C** Western blot showing the indicated proteins in Ctrl, PP-OE, and SRCIN1-OE + PP-OE U251 MG cells. The figures are representative data from at least three independent experiments. **D** Up: the expression of pY397 FAK, normalized to FAK, and the expression of pY416 Src, normalized to Src. Down: representative images of IHC analysis of indicated proteins in the intracranial tumors formed by PP-OE or the Ctrl U251 MG cells. The figures are representative data from at least three independent experiments. The data represent the mean ± SEM (*n* ≥ 3). Statistical analysis was performed using Student’s *t*-test. *P < 0.05, **P < 0.01, and ***P < 0.001.

To further explore the interacting protein with PPFIBP1, cell lysate of U251 MG cells was subjected to co-immunoprecipitation (Co-IP) using an anti-PPFIBP1 antibody with non-specific IgG as negative control, and the bound proteins were analyzed by liquid chromatography–tandem mass spectrometry (LC–MS/MS). Our Co-IP-coupled LC–MS/MS identified 201 proteins interacting with PPFIBP1, including SRCIN1 and LAMB1 (Supplementary Table 3). Intriguingly, our Co-IP assay showed that SRCIN1, but not LAMB1 bound PPFIBP1 (Fig. 5A). Downregulation of SRCIN1(P140Cap) enhances cell migration and integrin-dependent Src kinase activity [32]. So we next detected the expression of FAK and Src in GBM cells and tissues from xenograft. With western blot analysis, phosphorylation of FAK at Y397 and of Src at Y416 were found to be enhanced in PP-OE U251 MG cells, while abolished in shPPFIBP1 U251 MG cells (Fig. 5B). In addition, IHC analysis showed augmented phosphorylation of FAK and Src in PPFIBP1 overexpressing tissue from xenograft (Fig. 5D). Furthermore, overexpression of SRCIN1 rescued the expression of p-FAK and p-Src in PP-OE U251 MG cells (Fig. 5C). Together, these results suggest that PPFIBP1 may enhance the phosphorylation of FAK and Src through interacting with SRCIN1.

### Inhibitors of pSrc and pFAK reverse the pro-invasive caused by PPFIBP1 in glioma cells

**Fig. 6:**
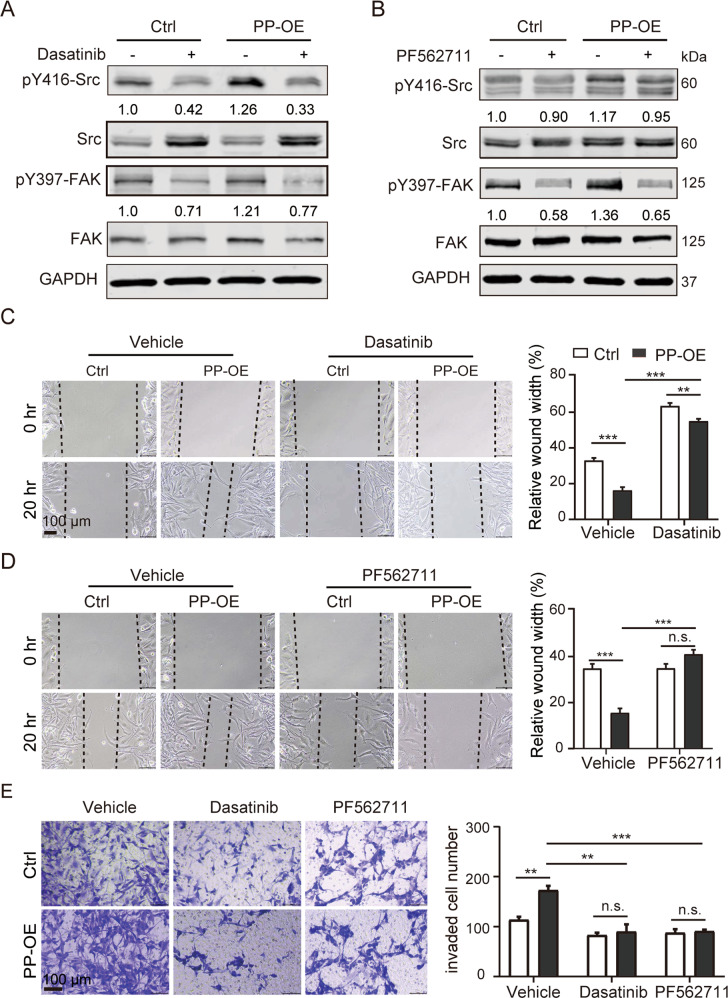
Inhibition of pSrc and pFAK reverse the promotion effect of PPFIBP1 on the migration and invasion. **A**, **B** Western blots showing the indicated proteins of U251 MG cells treated with or without 50 nM Dasatinib (**A**) or 1 μM PF562711 (**B**) for 30 min. The numbers represent the expression of pY397 FAK, normalized to FAK, and the expression of pY416 Src, normalized to Src. The figures are representative data from at least three independent experiments. **C**, **D** Wound-healing assays of U251 MG derived cells with PP-OE and the Ctrl at indicated time points. Left: representative bright-field pictures of cells at 0 or 20 h. Right: relative quantification of the (20 h/0 h) wound width. Dashed line represents the wound edge. Cells were treated with or without Dasatinib (**C**) or PF562711 (**D**) after scratches. **E** Transwell invasion assay with PP-OE and the Ctrl U251 MG cells. Left: Representative images of U251 MG cells stained with crystal violet. Cells were treated with or without 50 nM Dasatinib or 1 μM PF562711. Right: number of invaded cells through the membrane per field. The figures are representative data from at least three independent experiments. The data represents the mean ± SEM (*n* ≥ 3). Statistical analysis was performed using Student’s *t*-test. *P < 0.05, **P < 0.01, and ***P < 0.001.

To further validate the role of pFAK and pSrc in PPFIBP1-mediated migration and invasion of glioma cells, PP-OE and Ctrl cells were treated either with PF562711 (an inhibitor of pFAK) or Dasatinib (an inhibitor of pSrc). A series of concentration gradients of Dasatinib or PF562711 were used to treat the glioma cell lines, by western blotting, minimal effective concentrations of two inhibitors for each cell line were chosen (not shown). The results of western blot showed that 50 nM Dasatinib inhibited phosphorylation of Src and FAK and increased Src expression, while 1 μM PF562711 inhibited phosphorylation of FAK and Src (Fig. 6A, B). Inhibition of pSrc significantly reversed the migration and invasion velocity of PP-OE U251 MG cells (Fig. 6C, E). Similarly, inhibiting the activity of pFAK significantly restored the migration and invasion phenotype caused by PPFIBP1 overexpression in U251 MG cells (Fig. 6D, E). Together, these findings indicated that PPFIBP1 promoted glioma cell migration and invasion through FAK/Src pathway.

### PPFIBP1 activates JNK/c-Jun signaling in glioma cells

**Fig. 7:**
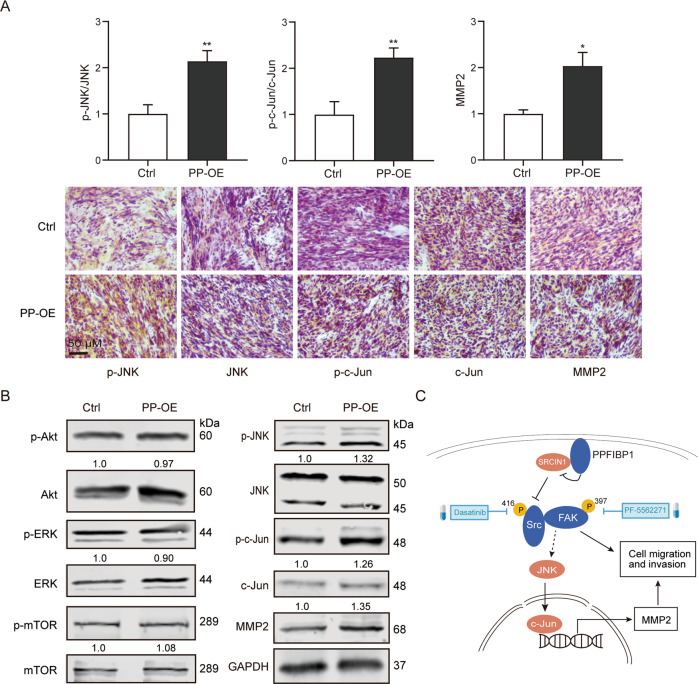
PPFIBP1 may promote MMP-2 expression through activation of JNK and c-Jun. **A** Up: the expression of pY397 FAK, normalized to FAK, and the expression of pY416 Src, normalized to Src, and quantification of other indicated proteins normalized to GAPDH. Down: representative images of IHC analysis of indicated proteins in the intracranial tumors formed by U251 MG cells with PP-OE or the vector control. The figures are representative data from at least three independent experiments. **B** Western blot analysis of indicated proteins in PP-OE and the Ctrl glioma cells. The numbers represent the expression of phosphorylation proteins normalized to total proteins, or quantification of other indicated proteins, normalized to GAPDH. The figures are representative data from at least three independent experiments. **C** A schematic representation of the proposed pathway by which PPFIBP1 promotes cell migration and tumor invasion in GBM cells. The figures are representative data from at least three independent experiments. *P *<* 0.05, **P < 0.01.

The FAK/Src signaling complex regulates several downstream signaling pathways including JNK/Jun [33], Akt/mTOR [34], extracellular signal-regulated protein kinase (ERK) [35], and small GTPases [36]. To determine whether FAK-enhanced cell invasion is associated with JNK/Jun, Akt/mTOR, and ERK in glioma cells, endogenous expression of these proteins was measured. An increased amount of p-JNK, p-c-Jun, integrins, and MMP-2 was detected in PP-OE glioma cells compared with the Ctrl cells (Figs. 5A, 7B). However, the expression of Akt, mTOR, ERK, and their phosphorylation showed no observable difference. Furthermore, IHC analysis also confirmed the increased phosphorylation of JNK and c-Jun in PPFIBP1 overexpressing tissue from xenograft (Fig. 7A). In addition, expression of E-Cadherin and N-Cadherin in PP-OE and the Ctrl glioma cells treated with or without PF562711 showed no difference (Figure S3). Therefore, the results indicated that PPFIBP1 might promote migration and invasion by stimulating expression of MMP-2 via activating of JNK and c-Jun in glioma cells.

## Discussion

GBM is the most aggressive brain tumor in human, while invasive growth is the leading obstacle to cure for GBM. Here we showed that elevated expression of PPFIBP1 positively correlates with higher tumor invasion and poor prognosis of glioblastoma patients. The transcriptional levels of PPFIBP1 in mesenchymal and proneural subtype were significantly higher than in classical subtype. The patient’s survival between high and low and PPFIBP1 expression group did not show statistic difference, indicating PPFIBP1 may not an independent factor to patients’ survival. We also provided evidence that PPFIBP1 promoted glioma cell migration and invasion in vitro, and increased glioma cell infiltration in the murine brain. Moreover, overexpression of PPFIBP1 also led to shorter survival of glioma-bearing mice. Further analysis showed that PPFIBP1 was associated with focal adhesion pathway. What’s more, we unravel a molecular mechanism that PPFIBP1 promotes human GBM cell invasion via FAK/Src/JNK axis.

Integrin signaling through FAK has been shown to promote cell motility in a number of studies [37, 38]. FAK is a non-receptor tyrosine kinase that plays critical role in signal transductions mediated by multiple cell surface receptors, including integrin [9]. In its inactive state, FAK forms a closed conformation with its FERM domain binding to the kinase domain [39]. In integrin-mediated cell adhesion, FERM domain interacts with the cytoplasmic domain of integrin-β1, which allows FAK autophosphorylation at Y397 and binds to Src family kinases [40]. The mutually activated FAK/Src complex then initiates multiple downstream signaling pathways to regulate multiple cellular functions such as cell migration and angiogenesis [9]. In the present study, we found that PPFIBP1 promoted the activation of FAK and Src. Moreover, suppression of pFAK and pSrc reversed the cell migration and invasion ability of PP-OE glioma cells. The results indicate that the promoting effect of PPFIBP1 on cell migration and invasion is at least partially through the activation of FAK and Src.

Previous studies have shown that integrin-mediated stimulation of FAK/p130Cas signaling pathways activates ERK1/2 and JNK1, and subsequently promotes MMP-2 secretion and cell invasion. The present findings indicated that PPFIBP1 stimulates activity of JNK and c-Jun, but not Akt/mTOR or ERK1/2 phosphorylation. Consistent with our result, it has been reported that JNK1 activation, but not ERK1/2 phosphorylation, is required in v-Src-stimulated FAK-Src-p130Cas signaling in FAK-null fibroblasts [35]. MMPs including MMP-2, which degrade extracellular matrix to overcome the extracellular matrix barrier at the invasive fronts of tumors, promote invasion of glioma cells into adjacent brain structures [41]. Our results also revealed that PPFIBP1 stimulates MMP-2 expression. Therefore, PPFIBP1 may promote cell invasion partially via FAK-Src-JNK-MMP2 axis in glioma cells.

SRCIN1 (p140Cap) is a novel Src-interacting protein and acts as a negative regulator of Src [42]. Increased expression of SRCIN1 leads to inhibition of Src and cell motility and invasion [43, 44]. Our results identified SRCIN1 as an interacting protein of PPFIBP1. Interestingly, overexpression of SRCIN1 rescued the activity of Src in PP-OE U251 MG cells. Thus PPFIBP1 competitive combination with SRCIN1 may lead to activation of Src. In addition, the unregulated activity of Src stimulates tyrosine phosphorylation of FAK at Y397 [9, 45]. The result indicated that overexpression of SRCIN1 rescued the activity of FAK in PP-OE U251 MG cells. Thus, PPFIBP1 may regulate phosphorylation of FAK and Src through competitive combination with SRCIN1. A more detailed mechanism needs further study.

Our results indicated that PPFIBP1 exerts a positive effect on GBM cell migration and increases expression of adhesion-related genes such as integrin family (ITGA3, ITGA4, ITGB8), KDR, PAK1, and VAV2. Whether different expression of these genes lead to the promotion of cell migration and invasion in glioma cells requires further exploration. Our results also revealed that PPFIBP1 was not associated with the epithelial to mesenchymal transformation (EMT) progress in glioma cells (Figure S3C).

To summarize our findings, we present a model (Fig. 7C) showing that upregulation of PPFIBP1 promotes FAK signaling and enhances MMP-2 expression via the FAK/Src/JNK axis may through interacting with SRCIN1, and subsequently enhances tumor cell migration and invasiveness. Our studies provide a basis for further characterization of the molecular cascades which are involved in PPFIBP1-stimulated glioma invasion. Although there is still no drug targeting PPFIBP1, many drugs are designed to target its downstream molecules such as FAK and Src kinase activity. Our results indicated that inhibiting the phosphorylation of FAK and Src reversed the migration and invasion phenotype caused by PPFIBP1 overexpression in vitro. Thus, PPFIBP1, FAK, and Src may serve as a potential target for anti-cancer therapy in glioma.

## Supplementary information

Supplementary data

Supplementary Table 1

Supplementary Table 2

Supplementary Table 3A

Supplementary Table 3B

Supplementary Table 3C

Supplementary Table 3D
