# Supplementary material for: PPFIBP1 induces glioma cell migration and invasion through FAK/Src/JNK signaling pathway
Source: Cell Death Dis. 2021 Sep 3;12(9):827. doi: 10.1038/s41419-021-04107-7 (PMC8417031; doi:10.1038/s41419-021-04107-7)
Supplement: Supplementary file 1 — Supplementary data [file 41419_2021_4107_MOESM1_ESM.docx]

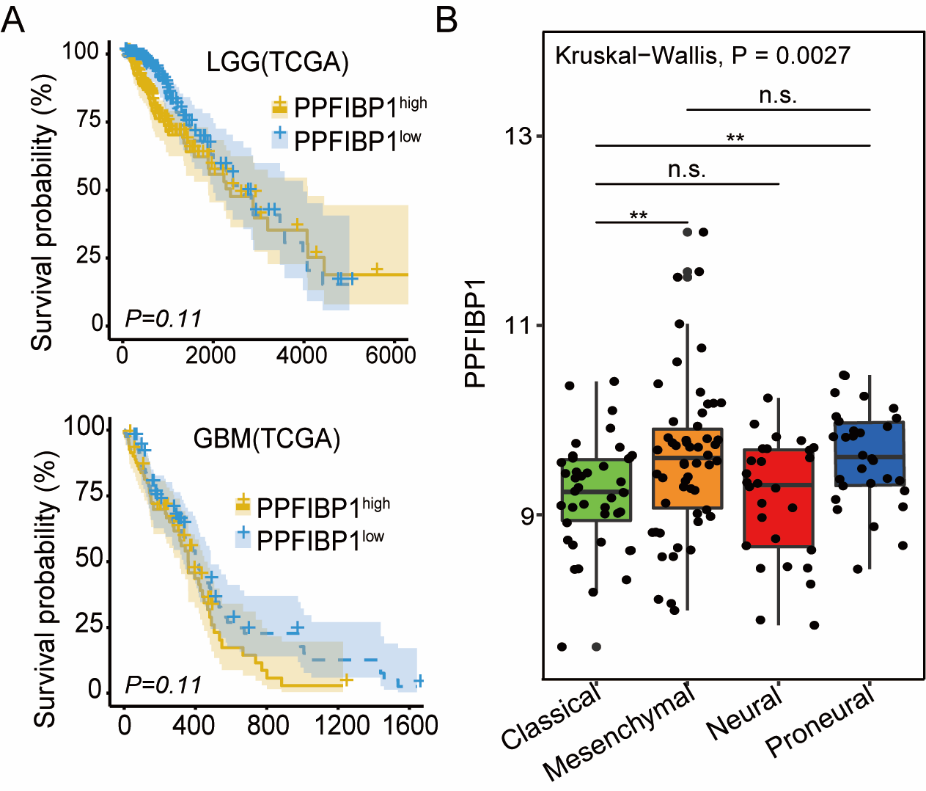


Figure S1. (A) Kaplan-Meier curves show the overall survival of 525 LGG and 165 GBM patients. Data was obtained from the TCGA database. (B)The boxplot shows the expression levels of PPFIBP1 in tumor tissue of patients with different subtypes. The dataset was from the same database as figure S1(A).


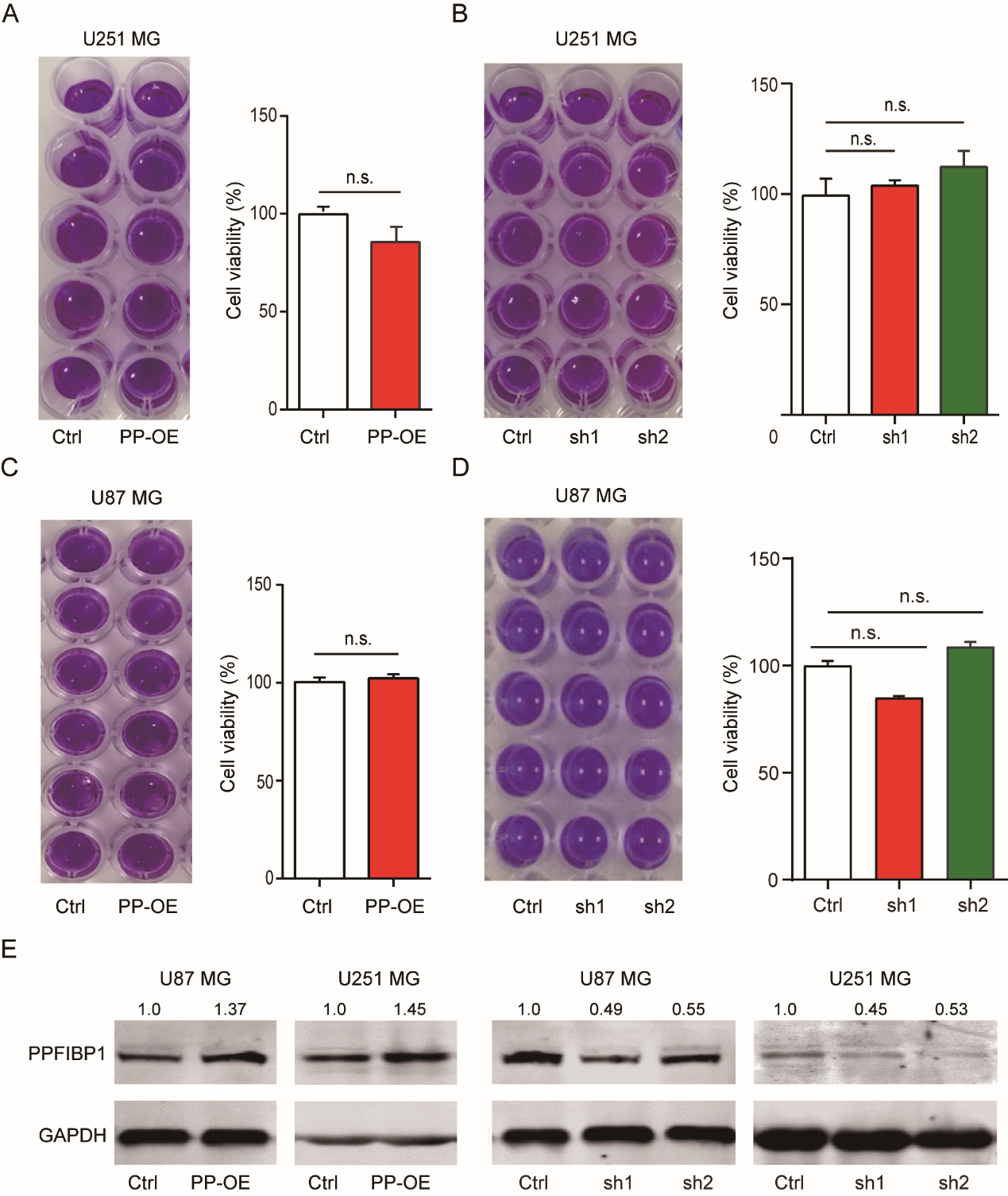


Figure S2. PPFIBP1 has little effect with cell proliferation of GBM. (A, C) Cell Titer Blue assay was performed with PPFIBP1 overexpressed U251 MG and U87 MG cells. (B, D) Cell Titer Blue assay was performed with PPFIBP1 silencing U251 MG and U87 MG cells. (E) Immunoblot analysis of PPFIBP1 in overexpressed and silencing U251 MG and U87 MG cells.


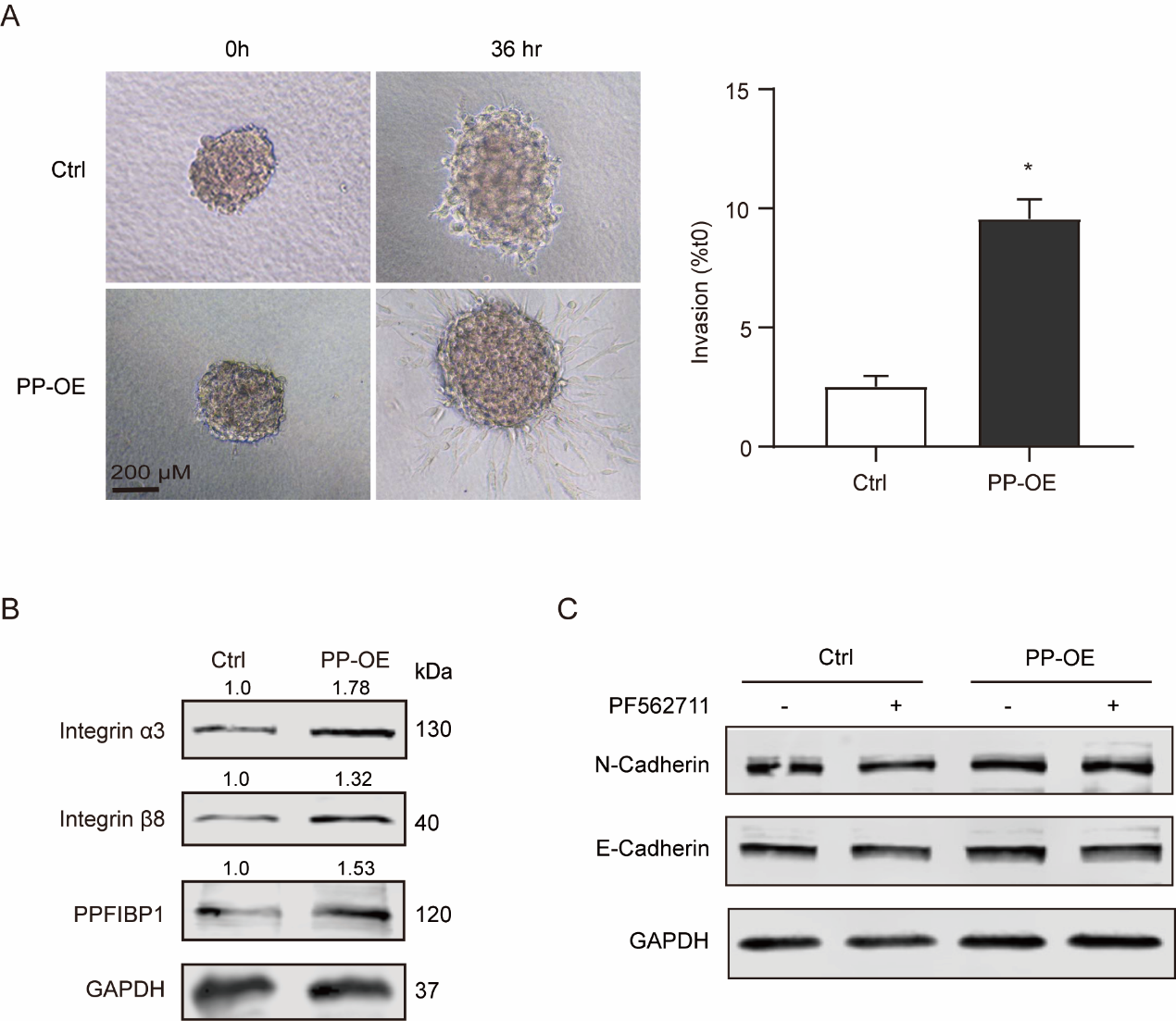


Figure S3. PPFIBP1 has little effect with EMT of GBM. (A) 3D spheroid invasion assay of U251 MG derived cells with PP-OE and Ctrl. (B, C) Western blot showing the indicated proteins in PPFIBP1 overexpression and the Ctrl U251 MG cells.
